# COMPARING EARLIER- VERSUS LATER-BORN CENTENARIAN COHORTS FROM THE HEALTH AND RETIREMENT STUDY

**DOI:** 10.1093/geroni/igad104.1340

**Published:** 2023-12-21

**Authors:** Gina Lee, Peter Martin

**Affiliations:** Iowa State University, Ames, Iowa, United States; Iowa State University, Ames, Iowa, United States

## Abstract

The current study aimed to compare the health status of earlier and later born centenarian cohorts. Using the data from the Health and Retirement Study, we selected those who survived to at least 98 years and divided them into earlier (n=189) and later (n=304) cohorts. The birth year of the earlier cohort ranged from 1890 to 1906, and the birth year of the later cohort ranged from 1907 to 1920. We first recoded variables to examine health status when all participants were at least 98 years old. To explore differences in health status between earlier vs. later cohorts, we computed t-tests/χ2 difference tests with activities of daily living, depressive symptoms, self-report of health and health change (SRHC), BMI, current smoking, drinking, cognition, and a number of diseases. There were significant differences in depressive symptoms, SRHC, high blood pressure, and current drinking between the earlier and later centenarian cohorts. The later cohort had significantly higher blood pressure, more depressive symptoms, worse SRHC, and later cohorts had a higher proportion of participants who currently consumed alcohol. Finally, the results of two separate regression analyses predicting depressive symptoms indicated that no demographic or health variables of the earlier cohort significantly predicted depressive symptoms, but years of education and current diagnosis of cancer significantly predicted depressive symptoms for the later cohort. Overall, the later born cohort had a worse health status compared to the earlier born cohort. This findings highlights the need for healthcare providers to pay attention to psychological health among cancer-diagnosed centenarians.

